# Viral apoptotic mimicry and the role of phosphatidylserine receptors

**DOI:** 10.1128/msphere.00089-25

**Published:** 2025-09-29

**Authors:** Melinda A. Brindley, Michael A. Pisciotta

**Affiliations:** 1 Department of Infectious Diseases, College of Veterinary Medicine, University of Georgia 1355, Athens, Georgia, USA; 2 Department of Population Health, College of Veterinary Medicine, University of Georgia 1355, Athens, Georgia, USA; Virginia-Maryland College of Veterinary Medicine, Blacksburg, Virginia, USA

**Keywords:** apoptotic mimicry, phosphatidylserine, TIM-1, TAM

## Abstract

In our 2021 mSphere of Influence article (M. A. Brindley, mSphere 6:e00034-21, 2021, https://doi.org/10.1128/mSphere.00034-21), we discussed the concept of viral apoptotic mimicry and how the seminal study by Mercer and Helenius proposed a new paradigm in how viruses can gain entry into cells (J. Mercer and A. Helenius, Science 320:531–535, 2008, https://doi.org/10.1126/science.1155164). Building on the observation that enveloped viral lipids can mediate viral entry, subsequent studies have expanded these observations and further defined the lipids that can initiate infection, the cellular receptors involved, as well as started to explore how viruses obtain their membrane and potentially alter the lipid environment to enhance infection. Exploring the role viral apoptotic mimicry plays in the host has proven more difficult, as many of the receptors that interact with viral lipids also play key roles in immune signaling. This Full Circle review summarizes the lipids and receptors that are involved with viral apoptotic mimicry, the viruses that use them, as well as examines the studies that attempt to explore the role apoptotic mimicry plays in a host.

## VIRAL ENTRY VIA APOPTOTIC MIMICRY

Viruses are obligate intracellular parasites that require host cells to replicate and transmit. To gain access to the host cell machinery, viruses must get past the plasma membrane to infect the host cell. Some viruses evolved to interact with specific cell proteins or carbohydrates to initiate entry. For example, human immunodeficiency virus (HIV) binds to CD4 on immune cells (1). CD4 binding induces a series of conformational changes in the viral glycoprotein ultimately enabling the viral glycoprotein to mediate fusion between the viral and host membranes (2, 3). These precise lock-and-key interactions between viral glycoproteins and cellular receptors can strongly govern host cell susceptibility and tropism. In 2003, Vanlandschoot and Leroux-Roels proposed that viruses might utilize the cellular pathway to clear apoptotic debris to gain access to the cell, now known as viral apoptotic mimicry (4, 5). Both professional and non-professional phagocytes contain receptors that recognize apoptotic debris, which when bound trigger endocytic engulfment or efferocytosis. Apoptotic debris is recognized primarily by phosphatidylserine (PS) content (6). PS is an anionic phospholipid typically present in the inner leaflet of the plasma membrane in healthy cells. However, when cells are damaged or programmed for elimination, caspase activation leads to PS exposure. Enveloped viruses can incorporate PS in their outer leaflet enabling them to hijack the cell’s natural mechanism to remove apoptotic debris and “trick” cells into engulfing the virus (7,8). These interactions are glycoprotein-independent, and several cellular receptors interact with PS, making this pathway more ubiquitous. The use of PS as a signal for apoptotic clearance is phylogenetically conserved (9); therefore, usurping this pathway enables entry into diverse hosts and can potentially facilitate cross-species jumps.

## APOPTOTIC CLEARANCE AND PS RECEPTORS (PSRs)

Efferocytosis of apoptotic debris is primarily mediated by professional phagocytes like macrophages; however, non-professional phagocytes also play a role (10). The debris is, in part, recognized by the high levels of outer leaflet PS in the membrane. Both professional and non-professional phagocytes contain various PSRs in their membranes. While PS is suggested to be the “eat-me” signal for efferocytosis, PS alone is not sufficient for engulfment (11). Apoptotic cells also produce “find-me” signals, soluble signals that attract phagocytes, and additional membrane changes occur, such as changes in carbohydrates and exposure of other phospholipids including phosphatidylethanolamine (PE), which facilitate clearance (12, 13). Because PSR binding to PS is not sufficient for apoptotic clearance, viral apoptotic mimicry may also require additional signals to initiate engulfment (14).

**Fig 1:**
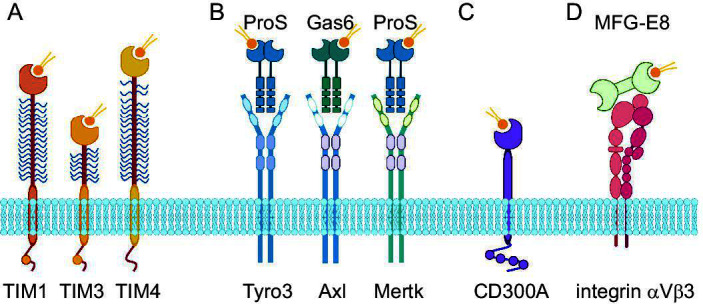
Phosphatidylserine receptors. (**A**) TIM family including TIM1, TIM3, and TIM4. The blue wavy lines represent the O-linked glycosylations making up the mucin domain. Both TIM1 and TIM3 contain phosphorylation sites within the cytoplasmic tails represented by a circle. (**B**) TAM family including Tyro3, Axl, and Mertk along with soluble Gas6 and Protein S (ProS). (**C**) CD300A, the ITIM motif is depicted in its cytoplasmic tail. (**D**) MFG-E8 and integrin αVβ3. For simplicity, the PS-binding sites are depicted with a single PS lipid. Created in bioRender.com.

PS recognition is critical for the proper removal of dead cells within a host and more than a dozen different PSRs are known (15–18). Some PSRs interact with PS directly, while other receptors bind to soluble PS-binding proteins, thus linking the phagocytes to PS-containing debris (Fig. 1). The T-cell immunoglobulin mucin domain (TIM) proteins contain a PS-binding motif that can directly interact with PS (19, 20); whereas the TAM family, made up of Tyro3, Axl, and Mer, bind to soluble PS-binding proteins Gas6 or Protein S (21). Additionally, CD300a, scavenger receptors, and brain-specific angiogenesis inhibitor 1 (BAI-1) directly interact with PS while milk fat globule epidermal growth factor 8 (MFG-E8) is another soluble factor that can bind to PS and link it to cells through interaction with integrins (12).

### TIMs

Humans encode three TIM family members: TIM1, TIM3, and TIM4. Structurally, all three are similar (Fig. 1A). The type-1 membrane proteins consist of an amino-terminal immunoglobulin variable (IgV) domain that contains a PS-binding site (19, 20). The IgV domain extends from the plasma membrane by a mucin domain. Dozens of O-linked glycans surround an extended protein chain forming a bottlebrush-like mucin domain of variable length (22). The extracellular domain is linked to the cell surface by a single-pass transmembrane domain and a short cytoplasmic tail. Mucin domains of both TIM1 and TIM4 are long enough to breach the cell glycocalyx, presenting the PS-binding domain above most of the other plasma membrane proteins. TIM3 is much shorter, resulting in its IgV domain sitting within the glycocalyx (22). While both TIM1 and TIM4 readily enhance enveloped virus entry, TIM3 did not, despite it containing a PS-binding site (23, 24). Chimeric TIM proteins containing TIM3 PS-binding domain/TIM1 mucin domains enhanced entry, while TIM1 PS-binding domain/TIM3 mucin domain did not (25), suggesting the IgV PS-binding domain needs to be above the glycocalyx to readily interact with virion PS. TIM3 can enhance apoptotic clearance (26), but in a cell-type-specific manner. This could suggest that in cells with a shorter glycocalyx layer, TIM3 is capable of binding to PS debris, but in cells with a thicker glycocalyx layer, TIM3 cannot mediate PS-binding. Both TIM1 and TIM3 contain tyrosine phosphorylation motifs in their cytoplasmic tails, yet TIM4 does not, suggesting TIM4 can only tether to PS while TIM1 and TIM3 can mediate signaling when bound to PS (27). It remains unclear whether TIM binding to virions is sufficient to initiate internalization. TIM1 lacking its cytoplasmic tail continued to enhance entry of vesicular stomatitis virus (VSV) pseudovirions coated in the Ebola glycoprotein lacking its mucin domain (ppVSV∆G + EBOV-GP∆O) particles (25). However, residues in the cytoplasmic tail were found to be critical for TIM1 enhancement of dengue entry (28).

TIM family members are associated with a variety of immune regulatory roles. TIM1 was first known as kidney injury molecule 1 (KIM1) and was found to be expressed on luminal epithelial cells within the kidney, especially after renal injury (29). TIM1 is expressed on T helper 2 (Th2) cells and is involved with T-cell activation (30). It is also found on other immune cells including B cells, dendritic cells (DCs), macrophages, invariant NKT cells, and mast cells (31, 32). TIM4 is produced in antigen-presenting cells (APCs) including macrophages and DCs (33, 34). When TIM4 on APCs and TIM1 on T cells both bind to PS-containing exosomes, it simulates T-cell proliferation (20, 35). Expression of TIM4 alone is not sufficient to clear apoptotic debris in several tissue macrophage lines and requires TAM expression to efficiently efferocytose (36, 37).

TIM1 was the first TIM family member associated with enhancing virus entry. In 1996, Kaplan et al. generated antibodies against African green monkey kidney cells that were susceptible to hepatitis A virus (HAV) (38). They identified three antibodies capable of inhibiting HAV infection that reacted with a mucin-like class 1 integral membrane protein that they named HAV cellular receptor 1 (HAVcr-1), now known as TIM1. Even though HAV is a picornavirus and lacks viral membrane proteins, the PS-binding domain of TIM1 is required for HAV entry (39). HAV and other “naked” viruses can acquire an envelope and travel from cell to cell in exosome-like vesicles (40, 41). TIM1 is needed for Vero cell uptake of these quasi-enveloped HAV particles (42).

In 2011, TIM1 expression was found to correlate with Ebola virus glycoprotein (EBOV-GP) mediated entry in a panel of human tumor lines (43). TIM1 monoclonals blocked both EBOV-GP and Marburg glycoprotein-mediated entry into several epithelial cell lines, suggesting TIM1 could serve as a receptor for filoviruses (43). However, TIM1 was not found in all susceptible cells; therefore, it was not required, and other factors could also mediate uptake. A gain-of-function cDNA library approach in 2012 found TIM3, TYRO3, and AXL enhanced dengue virus entry into 293T cells. Confirmatory studies found TIM3 modestly increased 293T susceptibility to dengue, while TIM1 and TIM4 were highly effective, increasing infection rates 500-fold (23). Interactions with both TIMs and TAMs occurred via PS in the viral particle (23). The following year, Jemielity et al. demonstrated entry of many enveloped viruses could be enhanced by both TIM and TAM family members and confirmed the enhancement is mediated by PS in the viral membrane (24). Subsequently, several studies have expanded the viruses that can utilize TIMs in entry (Table 1). Interestingly, human TIM1 is more efficient in mediating viral entry when compared to murine or rhesus TIM1 (44). Human TIM1 can bind to both PS and PE (45, 46), while murine and rhesus TIM1 only bind to PS (44), and the added ability to interact with PE may enable more efficient virus interactions.

**TABLE 1:** Studies that identify PSR in viral entry^*a*^

Viruses	TIM-1/4	TAM	MFG-E8	CD300a
Filoviruses	293T (24, 25, 43, 46–49) Vero (43) Huh7.5 (50)	Jurkat (51) Hela (50)		
Alphaviruses	293T (17, 24, 47, 52) HaCat (52) Huh7.5 (52) Vero (53)	293T (17, 21, 24)	HMVEC (17)	293T (17, 54)
Rhabdoviruses (VSV)	293T (17)	293T (17)		
Arenaviruses	293T (24, 55) Vero (55)	HT1080 (56) (29237830)		
Baculoviruses	293T (17, 47)	293T (17) HMVEC (21)	HMVEC (17)	
Flaviviruses	293T (23, 24, 46, 57, 58) Huh7.5 (28, 59) A549 (28, 57) 769P (28) Vero (60) RTEC (60)	293T (23, 58)		293T (54)
Poxviruses (vaccinia)		293T (21)		
Arteriviruses (PRRS)	MARC-145 (61)			
Asfarviruses (ASFV)		PAM (62)		
Coronaviruses (SARS-CoV-2)	293T (63)	293T (63) Vero, A549, H1650, HCC1944, HCC2303 (63)		
Retroviruses	293T (17)	293T (17)	HMVEC (17)	
Bunyaviruses (Hantan)	293H (64) A549 (64) Jurkat (65)	A549 (64)		
Picornavirus (HAV)	AGMK (66) Huh7.5 (67) LtK (38)			
HEV	Huh7.5 (68)			

^*a*^ Studies that exogenously produce the PSRs in cells are in plain text and include the cell lines used. Studies that either knock down a PSR or mask with PSR antibodies are underlined and include the cell lines used.

Most studies conclude that TIM1 and TIM4 facilitate virus entry by binding to the PS within the virion envelope (28, 69, 70). However, some studies suggest TIM1 directly binds to viral glycoproteins (71, 72). The results become difficult to interpret as the mutations in TIM1 identified to reduce binding to the glycoproteins are the same residues associated with PS binding (19, 71, 72). Furthermore, the same TIM1 residues associated with binding to Ebola and Japanese encephalitis glycoproteins, when mutated, also reduced VSV, Junin, and Lujo glycoprotein entry (73). Potentially, the PS-binding pocket also readily binds with proteins non-specifically. Additional studies, including control proteins, are needed to demonstrate that the binding is lipid-mediated.

### TAMs

Tyro3, Axl, and Mer (TAM) are a family of receptor tyrosine kinases primarily found on macrophages and DCs. Structurally, they contain two N-terminal Ig-like domains that mediate ligand binding, followed by two fibronectin type III repeats (Fig. 1B) (74, 75). The ectodomain is tethered by a single-transmembrane region, and the cytoplasmic tail contains a protein-tyrosine kinase domain. TAMs are involved in PS-mediated apoptotic clearance, but they do not bind PS directly; they bind proteins called growth arrest-specific-6 (Gas6) and protein S (ProS) (76). Gas6 and ProS are both secreted proteins that require vitamin K carboxylation to bridge TAMs and PS. The C-terminal domains of Gas6 and ProS bind to the TAM Ig-like domains, while the γ-carboxylated N-termini bind to PS (77). Gas6 and ProS’s affinity for PS is increased when PE is also present in the membrane (78). Gas6 and ProS do not interact with all TAMs equally. For example, Gas6 binds to Axl preferentially while ProS binds with Tyro3 (79).

TAMs are needed for apoptotic clearance in adult cells (80). In humans, it is estimated that more than a billion cells die every day, yet they are difficult to detect because macrophages and other phagocytes are so efficient in clearance (81, 82). Deletion of TAM receptors in mice results in the accumulation of dead cells due to inefficient clearance, which leads to male sterility and retinal diseases as well as autoimmune phenotypes from the build-up of self-antigens (83–87).

In addition to their role in apoptotic clearance, TAMs also play a role in innate immune signaling. Following engagement with PS-containing Gas6/ProS ligands, TAM receptor tyrosine kinases can dampen the innate immune response (88). For example, in DCs, Axl activation induces the downregulation of pro-inflammatory cytokines. Axl can form a complex with the type I interferon receptor (IFNAR) and switches signaling by activating suppressor of cytokine signaling (SOCS) 1 and 3 (88). TAMs are critical during innate immune signaling because if type 1 interferon binds to IFNAR, a pro-inflammatory signal is produced; however, if it binds to a TAM-IFNAR complex, an immunosuppressive response is produced. Since many viruses induce an interferon response, activation of TAM receptors will suppress the IFN signaling and may enable enhanced replication (89). TAM receptor activation can lead to cell survival and virus activation of TAMs could prolong the survival of viral-infected cells (90).

TAMs were first found to enhance entry of pseudotyped retroviral particles carrying the EBOV and MARV glycoproteins using a cDNA library approach (51). Follow-up studies confirmed TAMs enhanced viral uptake (91, 92), yet the mechanism did not involve interaction with the glycoprotein (93). Morizono et al. determined that Gas6 and ProS bind to virion PS and TAMs, which mediates enveloped virus internalization (21). TAMs' role in virus entry was confirmed by Meertens et al. with several additional enveloped viruses (23).

### Additional PS-binding proteins

Once the PS/PE in the viral membrane was demonstrated to be the ligand in apoptotic mimicry, studies to examine the role of other PS-binding partners were completed to determine if all PSRs worked in a similar manner. Moller-Tank et al. determined that not all PS-binding receptors mediated enveloped virus entry, as the receptor for advanced glycation end products (RAGE) did not enhance entry (47). Furthermore, Morizono and Chen tested whether MFG-E8, CD300a, BAI1, and stabilin 1 and 2 could facilitate PS-mediated viral uptake (17). MFG-E8 was found to work similarly to Gas6/Axl uptake, while CD300a could modestly increase uptake. However, not all PSRs enhanced virus uptake, neither BAI1 nor stabilins facilitated entry (17). While additional cellular proteins can interact with PS (12), little evidence suggests these other PSRs enhance viral uptake.

### CD300

CD300a binds to the aminophospholipids PS and PE. Human CD300a binds to PE with higher affinity than PS (94, 95), while CD300f (or mouse CD300lf) binds to ceramides and sphingomyelin in addition to PS (96). CD300 family members are found on many immune cells, including natural killer cells, DCs, and T cells. While CD300a binds to lipids exposed on apoptotic cells, it does not enhance apoptotic clearance (97). PS/PE binding to CD300a will negatively regulate the phagocytosis of apoptotic cells via an immunoreceptor tyrosine-based inhibitory motif (ITIM) within its cytoplasmic tail (Fig. 1C) (97). Despite reducing phagocytosis of apoptotic materials, CD300a enhances entry of dengue in 293T cells, and CD300a antibodies could partially inhibit dengue uptake into macrophages (54). CD300lf is the receptor for murine norovirus (98). While CD300lf binds to several lipids, including PS, TIM1 was unable to mediate norovirus entry, suggesting PS binding was not required (98).

### MFG-E8

 Milk fat globule-EGF8 (MFG-E8) is a soluble protein that binds to PS on the surface of cells and can initiate apoptotic clearance by linking to phagocytes through alpha(v)beta (1) integrin (Fig. 1D) (99). MFG-E8, also known as lactadherin, is abundantly found in milk but also produced in various other tissues. MFG-E8 was the first factor identified that could link apoptotic cells to phagocytes and contains 2 PS-binding domains (100). While MFG-E8 levels in milk can bind to rotavirus and prevent infection (101, 102), it was not associated with enhanced viral entry until Morizono and Chen found that it enhanced pseudoparticle entry into 293T cells (17). The integrin/MFG-E8/PS bridge helps HIV-1 virus-like particles spread in DCs (103).

## VIRAL APOPTOTIC MIMICRY

Once the first few studies showed that select enveloped virus entry can occur through PSRs, many additional reports were published demonstrating that the pathway can be used by numerous viruses, including Ebola, chikungunya, dengue, Zika, Tacaribe, and many others (Table 1). Most studies exploring the role of apoptotic mimicry and virus entry focus on taking poorly susceptible cells, overexpressing a PSR, and demonstrating viral entry is enhanced. For instance, 293T cells do not naturally produce PSRs, yet they are highly transfectable cells that can readily produce PSRs from exogenous plasmids (47). These enhanced entry observations have been documented for many viruses or viral glycoproteins (Table 1). While these studies demonstrate that overexpressed PSRs can bind and internalize PS-containing particles, they do not confirm that these pathways are used in naturally susceptible cells. A subset of studies knocked out PSRs from susceptible cells and demonstrated a large reduction in virus entry. For example, African green monkey Vero cells produce both TIM1 and Axl (104). A decrease or removal of TIM1 from these cells dramatically reduced entry of many viruses (28, 43, 48, 50, 52, 53, 55, 57, 59, 61, 64–68). The abundant levels of TIM1 and Axl may partially explain why Vero cells are frequently used for viral isolation. Vero cells readily uptake virions through apoptotic mimicry, and their lack of interferon production improves permissivity to several viruses (105).

Most studies characterizing viral apoptotic mimicry utilize viruses produced in various tissue culture cells. Moller-Tank harvested rVSV∆O/EBOV particles from several mouse tissues and found they readily infected cells producing TIM1 and virus entry was blocked by TIM1 antibodies or PS-containing liposomes, confirming host-derived viruses also become enriched in PS which can mediate entry (47).

Select studies demonstrate PSR requirements in disease-relevant cell types. Ebola virus is thought to target immune cells including macrophages during infection (106). While infection of various cell culture lines is attributed to TIM or TAM expression, monocyte-derived macrophages did not produce appreciable TIM1 or Axl, and they were not needed for infection, while integrin alphaV and Mer could mediate EBOV infection (50).

Bhattacharyya et al. hypothesized that the enveloped viruses may interact with TAMs not for entry, but because activated TAM receptors inhibit interferon signaling, and thus would be pro-viral (107). Using primary bone marrow-derived DCs from wild-type or TAM gene knockout mice, they determined that Gas6-bound virus rapidly activated Axl. Axl activation led to a dampened innate immune response, including type I interferons (107). They also found that blocking interferon with antibodies restores virus replication in TAM triple knockout cells, suggesting TAM’s role in dampening the immune response is more important than its role in viral entry (107).

SARS-CoV-2 entry into cells requires ACE2 (108), but in cells with low levels of ACE2 PSRs including TIM1, TIM4, and Axl can enhance entry (63). ACE2 expression in the respiratory tract is low, while many lung cells produce Axl (109). SARS-CoV-2 entry into several human lung cell lines was reduced by bemcentinib, an inhibitor that blocks Axl signaling (63), suggesting Axl may facilitate entry into disease-relevant cell types.

## HOW ENVELOPED VIRUSES OBTAIN THEIR MEMBRANE RICH IN PS

Cellular membranes are composed of phospholipid bilayers. The plasma membrane of a healthy cell is asymmetric, with distinct lipid compositions in each layer. The exoplasmic/outer leaflet is highly ordered and rigid, enriched in neutral phosphatidylcholine, sphingolipids, ceramides, and cholesterol. The tight packing is important to maintain the barrier role the plasma membrane plays, reducing permeability to extracellular material (110). In contrast, the inner leaflet is more fluid and contains amino-phospholipids PS and PE, as well as phosphatidylinositol (PI) (111). The net negative charge found on the inner leaflet serves as a platform to recruit many membrane-binding proteins to the inner leaflet of the plasma membrane (112, 113).

Plasma membrane asymmetry is critical for cellular function. To maintain distinct lipid organizations between the leaflets, cells produce families of lipid transport proteins, termed flippases and scramblases, that readily move lipids between leaflets (114–117). Flippases maintain bilayer asymmetry by actively flipping their lipid substrates found in the outer leaflet to the inner leaflet in an energy-requiring process (118). During specific signaling events, the asymmetric lipid organization can be briefly reversed, and inner leaflet lipids are exposed to the extracellular environment for short periods (119). Brief PS translocation is mediated by lipid scramblases, such as TMEM16F (120), which moves PS to the outer leaflet during myoblast fusion and exosome budding. Once the signaling event is complete, scramblases are turned off, and flippases restore membrane asymmetry. When cells are programmed for apoptosis, caspases cleave flippases, permanently inactivating them from lipid flipping. In addition, caspases activate scramblases such as XKR8, resulting in PS accumulation in the outer leaflet (121). Apoptosis induction results in an irreversible change to membrane asymmetry and will eventually lead to clearance by phagocytes. Some viruses manipulate cellular scramblases to alter the lipid distribution and enhance their infectivity, while others rely on the apoptotic changes that occur in the cell to increase PS on their membrane (8).

 For enveloped viruses to utilize apoptotic mimicry, they must incorporate PS and/or PE in their outer leaflet. As discussed above, cellular membranes are typically asymmetric, and PS/PE are only found in the inner leaflet of the plasma membrane. Therefore, virions that bud from the plasma membrane must alter the localization of lipids in the healthy bilayer in order to effectively produce virions that can utilize apoptotic mimicry. Many viral infections will eventually induce apoptosis, which will enhance the levels of PS in virions budding from the plasma membrane. Some viral infections activate scramblases, altering the membrane asymmetry and enhancing particle infectivity via PSRs. Ebola infection in Vero and Huh7 cells resulted in PS externalization via TMEM16F enhancing particle infectivity, while downregulation of the scramblase reduced particle infectivity (122). If cells lack the apoptotic-induced scramblase XKR8, the produced EBOV virions contain lower levels of external PS and are less infectious (53, 123, 124). Conversely, if the producing cells lack flippase complexes, viral particles become highly enriched in outer leaflet PS and can interact with PSRs more efficiently (53).

Viruses that bud from internal membranes can also incorporate PS and PE within their membrane to utilize apoptotic mimicry. Although PS is synthesized within the endoplasmic reticulum (ER), the ER membrane contains lower levels of PS than the plasma membrane. PS is also asymmetrically distributed with PS located in the luminal leaflet of the ER (125). While PS levels are low, PE is more abundant in the ER and Golgi, and viruses that bud from internal membranes may rely on PE interactions with various PSRs to help mediate entry. For example, flaviviruses including Zika (ZIKV), dengue (DENV), and West Nile (WNV) viruses bud within the ER (126). These viruses also infect and replicate in both mammalian and mosquito cells which have very different lipid levels (127, 128). Richard et al. determined Axl-dependent infection for flaviviruses could change depending on the cell type used to produce the virus (129). When the virus was produced in mammalian Vero 76 cells, ZIKV was able to readily infect human umbilical vein epithelial cells (HUVECs), while DENV and WNV were not (129). ZIKV entry into HUVECs was mediated by Axl, yet the closely related DENV and WNV viruses could not use Axl for entry. This was surprising, as previous studies had found DENV and WNV can use Axl for entry (23, 107). It was noted that previous studies produced DENV and WNV virus in mosquito C6/36 cells. When the experiment was repeated with C6/36 produced virus, DENV and WNV could utilize Axl. Lipid differences in ZIKV stocks also play a role in infecting immortalized villous trophoblasts and human placental explants; however, the role of specific receptors was not assessed (128). Mosquito-produced virus may contain altered lipid levels that facilitate Axl interaction.

 Entry of several non-enveloped viruses is enhanced by PSRs (40, 130, 131). While these viruses lack viral membrane proteins, they can utilize cellular exosome machinery to travel between cells (132). Exosome formation is enhanced in cells with high PS levels in the outer leaflet (133, 134). These quasi-enveloped viruses are enriched in PS/PE lipids that can facilitate uptake via PSRs. TIM1 expression of Vero cells was first identified as a viral entry receptor for the naked HAV (38).

 Virus binding to PSRs can facilitate entry, but cells producing PSRs on their surface can bind to newly budded virions and prevent their release, limiting virus spread. Several reports suggest PSRs reduce the release of newly formed HIV, Chikungunya virus (CHIKV), and Japanese encephalitis virus (JEV) (104, 135, 136). To efficiently spread and disseminate these viruses, actively reduce the levels of PSRs on the cell surface (104, 136–138). HIV’s nef protein reduces Axl levels on the cell surface, Japanese encephalitis virus NS2B-3 protein promotes the degradation of Axl, while CHIKV nsP2 protein reduces TIM1 expression (104, 136–138).

Few studies have examined how arboviruses such as dengue and chikungunya acquire PE or PS in the outer leaflet of their membranes within mosquito vectors. Perera et al. demonstrated that dengue virus infection of C6/36 cells leads to an altered lipidome, with significantly increased PE levels at sites of viral replication (139). In parallel, studies showing that dengue virus and West Nile virus (WNV) could utilize Axl in HUVEC cells only after passage through C6/36 cells (140) suggest that the altered lipid profile of insect cells is critical for PSR utilization and possibly for vector transmission. While homologs of known PSRs are not found in mosquitos, other PSRs are produced in insect cells and aid in apoptotic clearance (9), raising questions about species-specific roles of lipid-mediated entry mechanisms. The role of apoptotic mimicry in insect cells has not been robustly explored. However, CHIKV—which can use PSR for entry in mammalian cells—was found not to rely on PSRs in mosquito C6/36 cells, as entry could not be competitively inhibited by PC:PE:PS liposomes (53). This suggests that PSRs are not needed for CHIKV entry into C6/36 cells, but additional studies are needed to assess viral apoptotic mimicry in mosquito cells.

## THE COMPLICATED ROLE OF PSR IN ANIMAL MODELS OF DISEASE

Demonstrating that PSRs enhance viral uptake in tissue culture is clear; however, linking these interactions with outcomes of viral disease is complicated. The redundancy of PS-binding proteins makes it difficult to explore their roles in disease (141). For example, Zika infection is primarily mediated by Axl and Tyro3 in human skin fibroblasts, with TIM-1 playing a minor role (58, 140). However, mice deficient in Axl or Mer, or even double knockouts of both Axl/Mer or Axl/Tyro3, remained readily infectable with Zika virus after a subcutaneous inoculation (142, 143). Zika replicated to similar titers as control mice, suggesting removal of two associated receptors in the skin is not sufficient to alter mouse infection (142, 143). Additionally, similar results were observed after transplacental, vaginal, and intracranial routes of infection (143). Presumably, other PSR receptors like TIMs and/or CD300a (54, 78), or other non-PS pathways, facilitated mouse infection (143). In mice deficient for both interferon alpha receptor (IFNAR) and Axl, Axl played an age-dependent role in Zika-induced pathology (144). While all 3-week-old mice died from infection, 6-week-old mice lacking Axl were more likely to survive Zika infection due to reduced apoptosis in the brain (144). However, Mertk-deficient mice were more resistant to VSV because Mertk dampens the interferon response permitting more VSV replication (145).

TAMs play important roles in innate immune signaling, and while removal of a viral receptor may be predicted to reduce viral entry and spread, removal of important immune signaling pathways can play a more important role. Mice lacking Mer or Axl were more vulnerable to neuroinvasive West Nile and La Crosse viruses due to increased blood-brain barrier permeability (146). Axl-deficient mice were more susceptible to JEV, and more virus was observed in neuronal tissue, suggesting Axl’s role in altering immune pathways is more important than its ability to mediate entry within the mouse (147).

While the role of TAMs in animal models of disease suggests their immunologic functions are more important than their ability to enhance virus uptake, mice knocked out for TIM1 survived mouse-adapted EBOV significantly more than parental controls (148). Surprisingly, survival did not correlate with reduced viremia, as viral RNA copies in the blood were similar in TIM1^−/−^ and wild-type controls. However, viral levels were only noted in the blood on day 6 and no other tissues were reported. The lack of TIM1 reduced EBOV-stimulated cytokine production, and the authors suggest TIM1’s role in the T cell immune response to infection altered disease severity more than its role in virus entry (148).

TIM1’s role in viral entry was also monitored in a mouse model using a recombinant VSV encoding the EBOV glycoprotein (rVSV/EBOV). While Ifnar^−/−^ control and Ifnar^−/−^/TIM1^−/−^ mice both succumbed to rVSV/G infection, Ifnar^−/−^/TIM1^−/^− mice were significantly more likely to survive infection with rVSV/EBOV (149). Titers were examined over time and in various tissues following infection. While no differences were observed early in infection, by day 5, the TIM1^−/−^ mice had significantly lower titers in the serum, liver, kidneys, and adrenal glands (149), suggesting TIM1 may play a role in virus load. To examine the role of T cells in TIM1-deficient mice, mice were depleted of T cells and infected with rVSV/EBOV. T cell depletion did not alter mouse survival, suggesting that in this model of disease, T cells do not play a significant role (149).

TIM1 plays a significant role in mouse models of tick-borne encephalitis virus disease (60). Ifnar^−/−^ mice all succumbed to TBEV infection while 80% of Ifnar^−/−^/TIM1^−/−^ mice survived infection. Surviving mice showed little weight loss and lower viral titers than Ifnar^−/−^ that produced TIM1. Even in wild-type C57BL/6 mice lacking TIM1, they were significantly more likely to survive TBEV-Neudoerfl infection than wild-type mice. TIM1’s presence correlated with significantly more virus in the serum, brain, kidney, and lungs (60).

HAV results in mild or asymptomatic disease in most patients; however, a subset of patients develops serious hepatitis. Interestingly, HAV-induced severe liver disease is associated with an insertion in TIM1 which improves TIM1-HAV binding (150). The insertion adds six amino acids within the mucin domain of TIM1, and the insertion is also associated with HIV disease progression (151). While this association was observed in human cohorts, TIM1 did not play a role in entry into Huh-7.5 human hepatoma cells and mice lacking IFNAR1/TIM1 or IFNAR1/TIM4 remained susceptible to HAV infection and disease (42), suggesting TIM1 may play a limited role in HAV-induced disease.

## TARGETING APOPTOTIC MIMICRY FOR THERAPEUTICS

Rather than trying to reduce viral entry through genetic ablation of various PSRs, Soares et al. used an antibody against PS, bavituximab, to mask virion PS, preventing entry (152). Fifty percent of the guinea pigs infected with a lethal dose of Pichinde virus were protected if provided bavituximab 7 days following infection, and survival was improved if given in combination with the nucleoside analog ribavirin (152). Bavituximab also binds to the Ebola virus and EBOV-infected cells (153).

Rather than blocking the PS with an antibody, Song et al. engineered a soluble TIM domain (sTIMEdMLDR801). It was capable of binding to virion PS/PE and reduced viral infection in cell culture systems (154). The molecule slightly reduces Zika viral levels in a mouse model in the serum and spleen but did not alter levels in the brain, suggesting additional modifications will be needed for use as a therapeutic.

Bodily fluids such as saliva and semen are rich in extracellular vesicles (EVs), which can be antiviral (155–157). EVs are rich in PS, and EV production is enhanced in cells with high outer leaflet PS, suggesting EVs contain exposed PS (158, 159). Similar to lab-produced PS-containing liposomes, PS-rich EVs purified from human semen, saliva, urine, and milk were able to block Zika infection in Vero cells (160). The EVs from semen contained the highest levels of PS and were most effective in blocking viruses that utilize apoptotic mimicry including WNV, CHIKV, and EBOV-VLPs. Groß et al. noted viruses that readily transmit in bodily fluids such as HIV, herpesvirus, and hepatitis C virus do not use apoptotic mimicry. Many of the viruses that use apoptotic mimicry, DENV, ZIKV, CHIKV, EBOV, spread throughout the host in the blood, where EVs incorporate little PS (160). They suggest PS-rich EV-like particles could be developed into a therapeutic. Drugs or gene therapy formulated into lipid nanoparticles are seen to have increased effectiveness when the lipid species composition includes PS (161–164).

## FUTURE QUESTIONS AND PERSPECTIVE

** **Several outstanding questions remain regarding viral apoptotic mimicry. For example, some viruses utilize select PSRs, but not all. For example, SARS-CoV-2 entry is enhanced by Axl more than TIM1, while DENV entry is more efficient with TIM1 compared to Axl (23, 165). Are all PSRs interacting with the viral membrane in a similar manner, or are there virion differences that result in the selective use of PSRs? Are other cellular factors needed for different PSRs to efficiently internalize viruses? Does virion structure play a role in PSR usage? What is the role of apoptotic mimicry in the host? Can an effective, broadly active therapeutic be developed that effectively blocks viral entry via apoptotic mimicry?
